# Health-seeking pathway and factors leading to delays in tuberculosis diagnosis in West Pokot County, Kenya: A grounded theory study

**DOI:** 10.1371/journal.pone.0207995

**Published:** 2018-11-28

**Authors:** Grace Wambura Mbuthia, Charles Owour Olungah, Tom Gesora Ondicho

**Affiliations:** 1 College of Health Sciences, Moi University, Eldoret, Kenya; 2 Institute of Anthropology Gender and African Studies, University of Nairobi, Nairobi, Kenya; University of California San Francisco, UNITED STATES

## Abstract

**Background:**

Patients’ health-seeking behaviour can lead to delays in tuberculosis (TB) diagnosis, however little is known about the experiences and treatment pathways of patients diagnosed with TB in Kenya. The aim of this study is to explore the health seeking practices and factors contributing to delay in TB diagnosis.

**Methods:**

This study was based on explorative qualitative research using a constructivist grounded theory approach. A total of 61 TB patients in the intensive phase of treatment were recruited as informants in the study. Six focus group discussions and 15 in-depth interviews were used to collect data. Data were analysed through three step coding using the grounded theory approach.

**Results:**

Participants adopted different treatment pathways as they sought care from a pluralistic health care system involving traditional healers, herbalists, private clinic, drug shops and the public health sector. The study revealed an explanatory model of factors leading to delay illustrated by the participant’s expression “I suffered for a long time.” The model is comprised of three categories that lead to delays, namely individual, social-cultural and structural factors.

**Conclusion:**

There is a need to improve timely diagnosis of TB through innovative approaches such as intensive case finding. Similarly, the health-care system should decentralize TB services as much as possible and offer free diagnostic services to increase accessibility.

## Introduction

Tuberculosis (TB) is a global health concern due to its high morbidity and mortality rates. Globally, there were 10.4 million new (incident) TB cases and 1.4 million deaths in 2015. Kenya is among the 30 countries with the highest TB burden [1].

Delays in seeking TB treatment prolong the infectiousness of the disease, and therefore, early diagnosis of TB and rapid initiation of therapy is a prerequisite for the control of TB [2–7].

Studies have shown that delay in TB diagnosis and treatment is a critical problem in TB control programs which may have contributed to a high burden of TB in the African region [8–14]. As outlined in the WHO End TB Strategy, timely diagnosis and notification is one of the global priorities in the control of TB [15]. However, early case detection not only depends on the diagnostic capacity of the health facility but also the health seeking practices of the patients. In Kenya, as in the case of other low-income countries, TB control programs rely heavily on passive case finding. The programs depend on individuals with TB to voluntarily get help, and therefore patients’ health-seeking behaviour is critical to the success of these programs.

Health-seeking behaviour refers to the actions taken by individuals to enable them to treat or cope with their symptoms [16]. It is a complicated process [17] that entails patients’ interpretation of their symptoms and then making choices on treatment actions to adapt depending on the available options. Health-seeking behaviour is, therefore, a product of cognitive and non-cognitive factors that call for contextual analysis [18]. In West Pokot community, health care was pluralistic in nature with both the public and private health care system as well as traditional medicine. The traditional healer in this community was commonly referred to as *Chepsakitian*. According to the work of Kleinman on patients and healers in the context of culture, the health care system is pluralistic by nature with three closely overlapping sectors namely; the professional, the folk, and the popular sectors [19]. Kleinman contends that illness and disease is a social construction that is heavily influenced by the cultural beliefs of a community and that an individual’s experiences, attitudes, and health beliefs all influence a person’s health-seeking behaviour. Given the importance of culture in shaping patient decision-making, it is essential to understand the cultural and behavioural factors affecting TB control in order to inform patient-centred interventions in TB control [20–23].

Previous studies have documented health-seeking practices of TB patients in Kenya [20, 24]. However, none have focused on TB patients in the pastoralists communities. The seasonal movement in search of water and pasture for the livestock among nomadic pastoralists makes the population have the least access to health services which may lead to delay in TB diagnosis. Some of the documented factors leading to delay in TB diagnosis in Kenya are lack of finances to seek treatment and misinterpretation of early symptoms. Qualitative studies using grounded theory have not been applied to help understand the lived realities of TB patients in Kenya and specifically to understand their health care seeking behaviours. The purpose of this study is to understand experiences of health care seeking among TB patients and to generate a conceptual framework of factors leading to delay in TB diagnosis in West Pokot County.

Within Kenya’s devolved health care system there are four levels of public health care facilities under the control of county governments. These are County referral hospitals, Sub-County hospitals, health centres and dispensaries. The health centres and dispensaries offer primary health services while the County hospitals and Sub-county hospitals offer curative services and have inpatient facilities and better diagnostic capacity. TB diagnosis in West Pokot County followed national guidelines [25] where the standard test for TB was sputum smear microscopy. In Kenya, sputum microscopy services are available at the health centres, Sub-county hospitals, and County hospitals but not at the dispensaries. Clinical diagnosis was also made at the County referral hospital through the aid of chest radiograph. Diagnosis using cultures were not available. The hospitals mainly relied on microscopy for TB diagnosis, one X-ray machine and one Gene X-pert machine located at the County referral hospital.

## Methods

### Study setting

This study took place in four hospitals offering TB services in West Pokot County, Kenya. The County is located in the Rift Valley and lies within Longitudes 34° 47´ and 35° 49´ East and Latitude 10° 10´ and 30° 40´ North. The County has a population of 512,690 people (2009 census) and an area of 9,169.4 km2. The residents are mainly nomadic pastoralists living in the arid or semiarid part of the country. West Pokot County had a higher TB case notification rate of 225 per 100,000 population compared to the national rate of 217 per 100,000 population in the year 2013 [26]. The study was carried out in four facilities which included 3 Sub-County hospitals and the County referral hospital.

### Study design

This was explorative qualitative research using a constructivist grounded theory approach [27].The theory assumes a relativist ontology of multiple possible social realities and recognises that knowledge is co-constructed by the researcher and the research participants. Constructivist grounded theory aims to achieve an interpretive understanding of the research subjects’ meanings [28]. Grounded theory was used to generate a model of the factors leading to TB diagnostic delays from the patients’ perspective. The study adopted a constructivist approach where both deductive and inductive reasoning was used to interpret the participant’s views and experiences [28].

### Participant recruitment and sampling

The participants composed of 61 newly diagnosed pulmonary TB patients who were receiving treatment from at least one of the four health facilities in West Pokot County. The study was conducted between December 2015 and March 2016. The participants were recruited through the help of the nurses working at the various TB clinics. The nurse assisted in identifying patients who met the inclusion criteria. Only confirmed TB patients in the intensive phase (first two months) of TB treatment were included in the study. Due to the infectious nature of the disease, patients who had not completed two weeks of treatment were excluded from the study. The researcher briefed the identified patients about the study by taking them through the informed consent form. Participants willing to take part in the study were asked to sign the consent forms and were booked for interviews at the time of their appointment dates.

### Data collection methods

#### In-depth interviews

In-depth interviews (IDIs) were the primary method used to collect data on patient experiences and treatment pathways experienced during TB diagnosis. The interviews were conducted by the principal investigator and two research assistants. The research assistants were master’s students who had training in research skills and the PI orientated them to the study objectives and the study tools. The IDIs took place at the TB clinics and lasted for 60–90 minutes. A semi-structured interview guide based on the research questions was used to collect the information. The guide was adjusted in the subsequent interviews to probe on new emerging themes from the earlier interviews. The interview guide included questions such as: “Tell me about the experience you have gone through since you started ailing from TB? What actions did you take when you started experiencing TB symptoms (the health seeking practices in chronological order)?” “Why do people delay before seeking TB treatment from the health facility?”

The interviews were conducted in Kiswahili language and were tape recorded and later transcribed verbatim [29]. The concept of data saturation [30] was used to guide the number of IDIs conducted. Data collection was stopped at 15 IDIs. The Kiswahili tape-recorded interviews were translated into English and transcribed in English. The audio records were stored under key and lock, and the transcribed data was stored on a password-protected computer.

#### Focus group discussion

We conducted six focused group discussions (FGDs) (Three with male participants and three with female participants). Each group comprised of 6–10 participants. The FGDs were constituted based on gender. The principal investigator facilitated the discussion as the research assistant recorded notes during the discussion. An unstructured FGD guide was used to collect the data. The guide was revised in the subsequent FGDs to probe on new emerging themes. The guide included questions such as; “What are some of the traditional explanations to the causes of TB?” “Tell me about the experience TB patients go through to get TB diagnosis and treatment?” “What other forms of health care for TB patients is available in this community?”

FGDs were meant to complement the IDIs. Compared to individual interviews, group interaction allows participants to agree and disagree thereby stimulating richer responses which aid in revealing the respondent’s real perceptions on the subject of interest [21]. Gathering ideas and cultural beliefs surrounding TB was possible through this method of data collection. Participants gave more information on cultural practices in TB diagnosis and treatment during the FGD compared to the IDI participants. FGD participants facilitated each other’s memories and were more willing to report their experience of traditional medicine compared to IDI participants. This could be due to fear of being disapproved by the peers.

The FGDs were conducted in Kiswahili language, and each lasted for 60 to 90 minutes. The FGDs were audio-recorded and later transcribed verbatim [29]. The concept of theoretical saturation was used to ensure no new conceptual information was emerging from further discussion [30]. Data saturation was reached at 6 FGDs. The Kiswahili tape-recorded discussions were translated into English and transcribed in English.

### Data processing and analysis

**Fig 1:**
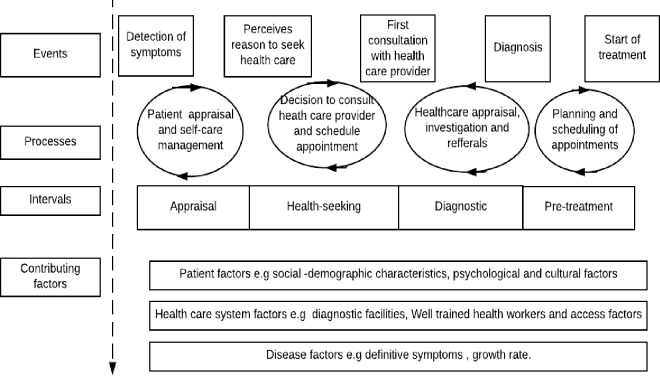
The model of pathways to treatment according to Scott et al. (2013).

The focus group discussions and in-depth interviews were transcribed and coded by the principal investigator. The transcripts were analysed with the aid of the N-vivo (version 11). Data collection and data analysis were done concurrently. During this process, the researcher wrote memos and reflections after each IDI /FGD. The treatment pathway model shown in Fig 1 [31] informed the current study. The model outlines the events, processes, and intervals that occur before diagnosis and treatment commencement.

The treatment pathway framework acknowledges that health-seeking is not a similar sequence of events, but instead, there are many possible routes to diagnosis that are influenced by various factors and can lead to delay in diagnosis [31]. Information on different routes to diagnosis and patients experience during the different intervals and process of health seeking was key in the study. Different factors contributing to the health seeking practices adopted were also examined. Theoretical sampling was done by formulating probing questions that would be asked in the consequent FGD or IDI to clarify emerging concepts.

An analysis was done by reading the transcript multiple times and identifying, coding, and categorising meaningful patterns into themes and subthemes using a constructivist grounded theory approach. A constructivist grounded theory considers that the researcher comes into research with existing assumptions from literature but makes an effort to analyse and develop the theory from what is emerging from the data [27]. The analysis incorporated the three levels of coding used in grounded theory analysis: open, axial and selective [32]. Open Coding was done by reading the transcripts word for word many times and identifying codes from the data. Related codes were grouped into sub-categories during axial coding. The subcategories were then integrated into theoretical constructs in the selective coding. The axial and the selective coding were used to explore the relationships between the subcategories and tie the concepts together to generate a conceptual framework through comparative analysis [32].

### Reflexivity

GWM, a Ph.D. student in Medical Anthropology and a registered community health nurse working in nurse education in Kenya, with two trained research assistants, conducted the research. Being a health worker researching in Kenya was likely to influence the interpretation of the findings. According to Carter 2007, a qualitative researcher should observe the real attitudes, motivations and beliefs of the participants and avoid introducing bias in the study [33]. Efforts were made to set aside prior knowledge of TB and to present the views of the participants and not those of the researcher through bracketing [34]. To ensure patients felt comfortable discussing their experiences GWM introduced herself as a student researcher who worked in a different region in the country and whose role was only research related. The health worker at the TB clinic helped in the identification of the patients to take part in the study but was not allowed to sit in during the interviews to allow participants to express themselves freely. TO and CO established anthropologists at the University of Nairobi Kenya gave guidance during the design, data collection as well as analysis.

### Ethical considerations

The research proposal was approved by Moi University- Kenya/Moi Teaching and Referral Hospital Institutional Research and Ethics Committee (Formal Approval Number: IREC 0001349). Participants were briefed on the purpose of the study, and each participant asked to sign an informed consent form without coercion. Participants allowed the audio recording during data collection and were assured of confidentiality and anonymity for any information given.

## Results

### Socio-demographic characteristics of the participants

A total of 61 participants took part in the qualitative data collection. This included 22 females and 24 males for the focus group discussions and seven females and eight males for the in-depth interviews. Their age ranged from 27–61years.

### Health-seeking pathway

**Fig 2:**
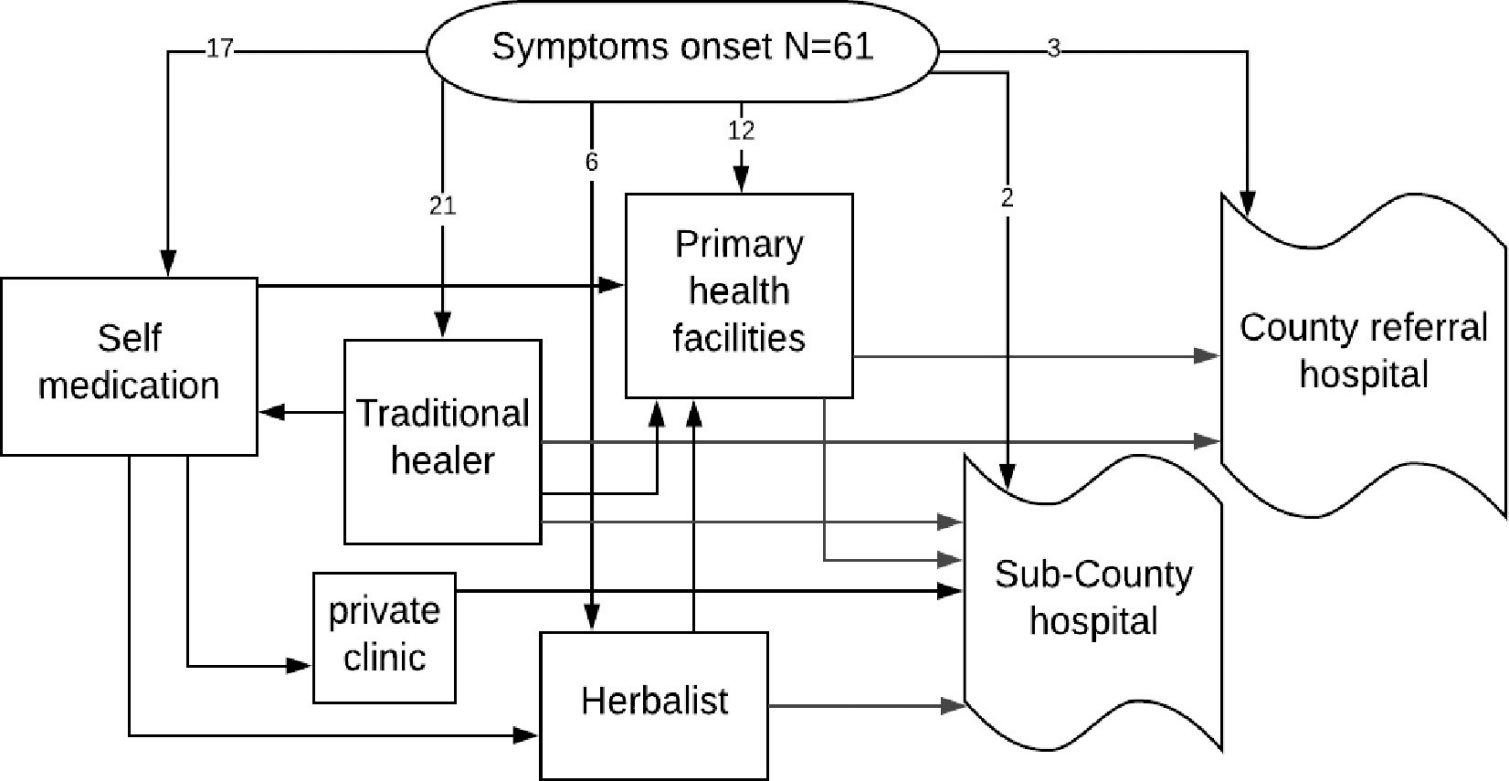
Schematic model of main treatment pathways followed by patients in West Pokot County.

Participants reported having sought care from different providers. The main health-seeking pathways adopted by the patients are as illustrated in Fig 2. The figure shows there were multiple actors in the provision of healthcare and participants had different choices as their first points of care. A third 21(34%) of the participants chose traditional healers, 17(28%) self-medication, 12(20%) primary health facilities, while 6(10%) of the participants opted for herbalists as their first point of care. Only 5(8%) of the participants visited either the Sub-County or the County hospital in the initial visit.

Participants reported different health-seeking trajectories and switched from one provider to another in search of relief of symptoms. One participant noted:

“In my case, I was sick for almost two years. I tried many things I bought medicine in the shops, I went to the local medicine man who gave me some herbs, but this did not help. I also went to the dispensary many times, but they treated me for other things. I had a severe cough for two months after which I came here, and my sputum was tested, and they found TB.” *(*2^nd^ female FGD).

After experiencing symptoms participants often resorted to self-medication from drug shops before seeking another form of care.

“And you know when this disease sets in you will start coughing continuously so you don’t stop and you buy drugs from the chemist now and then because you are always ill. You keep changing the drugs, but you realise you are not getting well.” (IDI Female 36 years).

Some (34%) of the participant's believed that illness was rarely caused by natural causes alone. According to the participants, illness was caused by bad omens evil spirits and witchcraft and such illness could only be treated by a traditional healer (commonly known as *Chepsakitian* among the Pokot community).

“… You know most severe disease comes as a result of curses and witchcraft; it is only Chepsakitian …who can cure you of such.” (2^nd^ female FGD).

For this reason, the participants visited the traditional healer first before seeking care from modern health facilities.

After undergoing the traditional therapy and noticing no improvement, participants tended to seek care from health facilities which were either primary health care facilities or Sub-County and County hospitals depending on the severity of the illness. This pathway was illustrated as follows:

“…at first I thought it was just a normal cold and so I didn’t take any action. But after some time the cough increased, and I decided to seek treatment. I went to the local herbalist… But after taking medicine for a long time, I realised the problem was persisting. When I realised it was not helping I came to the hospital.” (IDI Female 39 years).

When self-treatment failed, some participants sought treatment from private clinics. However, none of the participants reported having been diagnosed with TB from the private clinics. It was only after developing severe symptoms that the participants sought treatment from either the Sub-County or the County hospitals where TB diagnosis was made.

“I had started coughing long before, but I only bought medicines from the shops. I did not improve, and when it got worse I went to a private clinic in January where I was told that it was Pneumonia… But my illness got worse … I therefore decided to seek treatment in a bigger hospital and came to… hospital… and I was found to have TB.” (IDI Male 27 years).

For majority 39(63%) of the participants, a primary health facility was their first contact with the public healthcare system. However, they were not diagnosed with TB at these facilities, and all ended up at the Sub-County and County hospitals where they finally obtained a TB diagnosis. At the primary health facilities, patients were mainly treated for upper respiratory tract infections, typhoid, and malaria.

“I have suffered a lot with this illness …I went to the dispensary and was given medicine, but I did not get well. I went back to the same dispensary and was given another medicine, but still, I didn’t get well. After I got very sick, my children took me to … county hospital and there I was tested for TB, and it was negative….they tested me again they said I had TB.” (IDI Male 45 years).

The varied treatment pathways resulted in delay in diagnosis due to multiple factors discussed in more detail in subsequent sections.

### A conceptual model of factors associated with the delay in diagnosis

**Fig 3:**
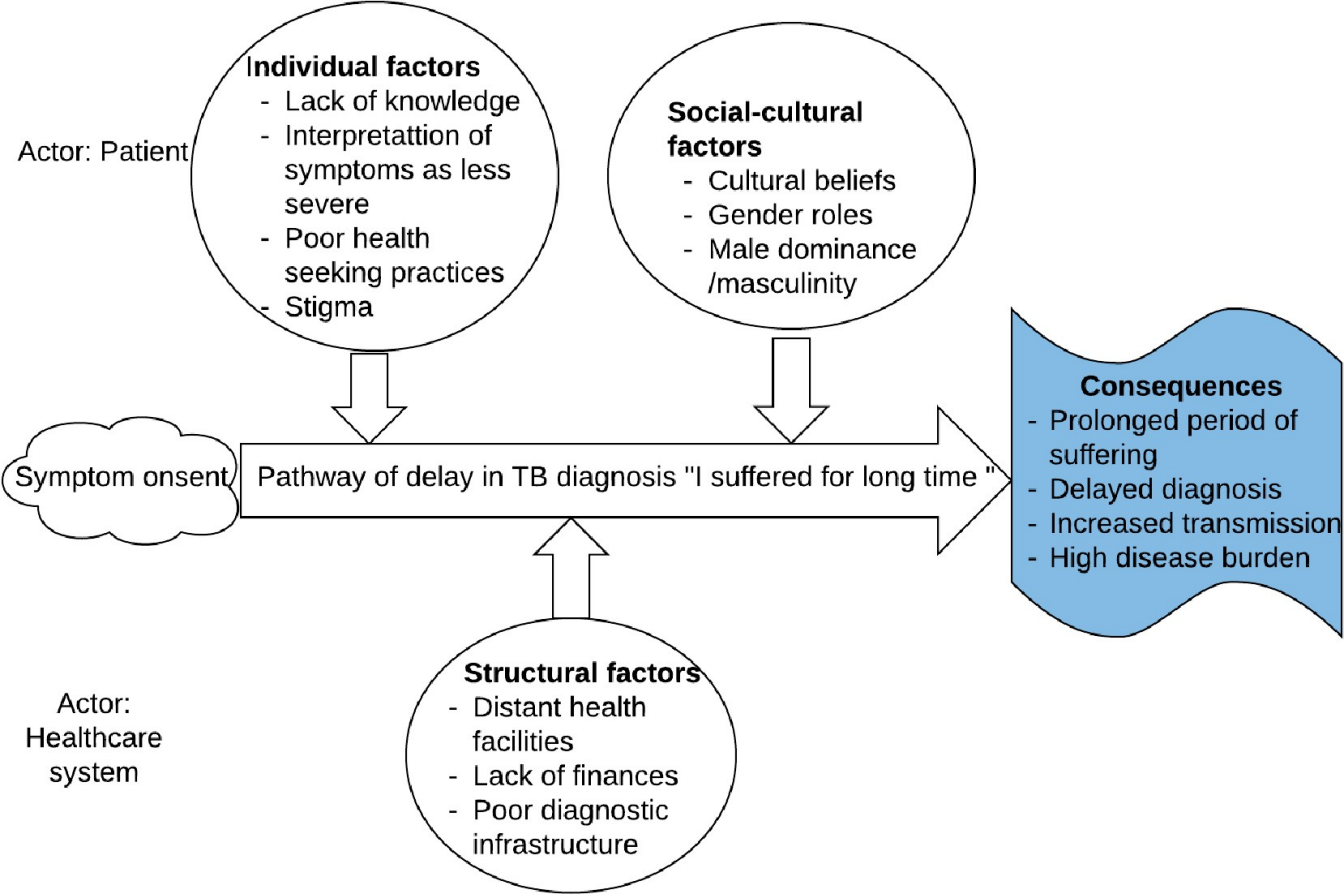
Conceptual framework of factors leading to delay in TB diagnosis in West Pokot Kenya.

A total of 17 codes emerged from the data. The related codes were then grouped into three major themes (categories) [31]. The in vivo code “I suffered for a long time” was a subcategory which was expressed by the participants as a summary of their experience of seeking TB diagnosis. It was used to link the three themes and formed the core category in the pathway of delay. The health-seeking experiences of the patients can be summarised in a conceptual framework of a pathway of delay that illustrates the factors that lead to delayed TB diagnosis in Kenya (Fig 3). The framework demonstrates three categories of factors that influence patients’ health-seeking practices namely: individual, socio-cultural and structural factors. The consequences of the pathway are delayed diagnosis, increased transmission and prolonged period of suffering.

#### Individual factors

As shown in the framework, individual factors include lack of knowledge, interpretation of symptoms as less severe and poor health-seeking practices.

Lack of knowledge was a contributing factor to people turning to alternative medicine and only presenting to the health facilities after severe symptoms set in. Participants argued that there was no need to seek hospital treatment following symptoms onset since good alternative medicine was available locally. This was alluded to as follows:

“If you go to the markets you can get good local herbs. They help a lot. When you start coughing, you don’t run to the hospital immediately. One should go to the hospital when the illness gets worse.” (1^st^ male FGD).

The participants also interpreted symptoms as less severe. After experiencing TB symptoms, the majority 43(70%) of the participants reported that they did not seek immediate treatment since they assumed this was a normal common cold that would resolve on its own. Others attributed the cough to the dusty environment and smoking. It was only after experiencing serious symptoms such as coughing blood, severe chest pain, that they sought TB treatment from the hospitals.

“I had coughed for almost one year. I thought it was just a normal cough that will go away…when I started coughing blood, I realised the disease might finish me. That is when I decided to come here.” (2^nd^ female FGD).

“At first it started just like a normal cough which I assumed. I used to think it is a cold or it was as a result of my smoking.” (IDI Male 32 years)

Similarly, poor health-seeking behaviour and alternative medicine resulted in substantial delay. After experiencing TB symptoms, patients opted for other forms of treatment before seeking treatment from a health facility. Some of the health-seeking practices among the community members included: self-medication, herbal medicine, traditional healers and religion.

“Like for me, the whole year it was a matter of buying Amoxil because that malaria had affected the chest, that malaria is bad.” (2^nd^ female FGD).

“I used the herbal drugs for a period of one year. You know they are of different types from different trees. So what we do we try this one when it fails you try the other.” (IDI Female 39 years).

Patients indicated that they preferred herbal medicine since it was easily available. They argued that, in earlier generations local herbs were used successfully to cure diseases before conventional medicine was made available. According to them, the herbs have no side effects.

“The reason people prefer the traditional medicine to modern medicine is that the former is more accessible and cheaper than the latter. Before you can get the money to travel to the hospital you want to try something that is available close to you.” (3^rd^ female FGD).

“…These do not have side effects like the medicine from the Whiteman. The time of our grandparents the medicine we have today was not there, but people still used to be treated. But right now we end up in hospitals to take modern medicines.” (3^rd^ male FGD).

Despite having used different forms of traditional medicine, the participants noted that they ended coming to the health facility for TB treatment.

“Here many herbalists will give all sort of medicines. Sometimes they help but other times they don’t. We try them, but we end up coming to the hospitals.” (2^nd^ female FGD).

#### Socio-cultural factors

Social-cultural factors were also key in influencing the time taken from the development of symptoms to the time the patient sought health care. These included; cultural beliefs, gender roles, masculinity and male dominance.

Participants’ cultural beliefs influenced their health seeking behaviour. They reported that, after experiencing TB symptoms, they first visited the traditional healer before seeking medical treatment. The community believed that an illness which is as a result of evil spirits could lead to death when treated with contemporary medicine. In one of the focus group discussions, all members admitted having visited the *Chepsakitian* before seeking modern medical care. Some of the participants pointed that:

“They are very many. Almost in every village, you will find one. And most people before going to the hospital they have to go through the traditional doctor before going to the hospital. Our community believes that for many diseases, there are evil spirits that bring the problem and so before even seeking hospital treatment it is good to remove this spirits for better results… Everybody here must have gone to Chepsakitian.” (2^nd^ female FGD).

Gender roles, masculinity and male dominance contributed to delays in TB diagnosis. The males were seen as the heads of the family and were supposed to provide and take control of the family. Females assumed submissive roles, and they were not supposed to go to the hospital without the husband’s approval.

“You have to ask for permission. You can’t go like that… They don’t see the need for us to go to the hospital.” (2^nd^ female FGD).

According to the participants, male patients lack time to seek health care as they are busy earning their daily living.

“…but with us men when you fall sick with a minor problem like coughing we don’t act fast. We usually persevere first because you have other things to do to earn a living.” (IDI Male 37 years).

Male dominance was a key factor contributing to delay in seeking TB treatment amongst female patients. In addition to the fact that females had to seek permission from their male partners before going to the hospital, they were also economically disadvantaged as they relied on the male partners to provide finances to seek care. This was not granted freely therefore resulting in delays.

“We quarrelled with him several times until he gave me money for transport. I told him to allow me to go and have my chest checked because it had deteriorated.” (1^st^ female FGD).

The participants reported that masculinity was a hindrance to the timely management of TB. For the males, the act of seeking immediate medical attention was seen as an act of weakness. They were supposed to persevere and only seek treatment when the condition is severe. This was alluded to by several participants:

“…that time it was not very bad. As a man, you don’t fall sick today, and tomorrow you are in the hospital. We go to the hospital when it is very severe.” (IDI Male 32 years).

#### Structural factors

Structural factors included distant health facilities, lack of finances to access the healthcare as well as poor diagnostic infrastructure that results in misdiagnosis.

When asked why they delayed seeking treatment for TB symptoms, some of the patients reported that being far from a health facility was the main barrier to seeking treatment. One of the female participants said:

“Some of us come from far, and there is no hospital nearby, even the dispensary is very far, and you find you have no fare to get to the hospital and you are already sick and are supposed to go get the drugs. Although the TB treatment is free in the government hospital, we still encounter a lot of challenges.” (1^st^ female FGD).

Similarly, some of the participants indicated that lack of money to pay for transportation and investigations required at the health facilities was a hindrance to prompt care seeking. Some of the participants resorted to local herbs or self-medication which to them was more accessible. When asked why he delayed seeking care from the hospital one male patient pointed that:

“The problem was I didn’t have fare to go to the hospital.” (IDI Male 32 years).

The participants agreed that poor diagnostic infrastructure was one of the main factors contributing to delay in TB diagnosis. The majority 51(83.6%) of the participants had a misdiagnosis. According to them, getting a TB diagnosis was a frustrating experience. Even after the patient had sought healthcare, they were treated for other ailments other than TB. Most patients were treated with antibiotics for pneumonia or upper respiratory tract infection. This led to a delay in diagnosis.

“They couldn’t realise I had TB; I suffered a lot. I went from one hospital to another and still could not find TB. You know TB is a bad disease. It hides in the body, and it finishes someone slowly by slowly. The first time I went to the hospital and was told that I had pneumonia, then meningitis. I was given medication, but the cough did not stop. I was treated for the wrong disease.” (IDI Female 30 years).

Misdiagnosis was reported mainly in the dispensaries and the private clinics. Those who sought care at the dispensaries and private clinics were treated with antibiotics and were neither offered a sputum test nor referred for sputum test. Repeated visits to the dispensaries led to delayed TB diagnosis. It was only after the participants noticed severe symptoms that they sought treatment at the Sub-County / County facility where they were offered a sputum test and chest x-rays. This was alluded to by one of the FGD participants as follows:

“When I feel sick I went to the dispensary and was given some drugs which never helped me. I kept going back to the dispensary, but they never checked my sputum. I later went to a private clinic where I was given injections, I improved a little, but later I got worse and came to the hospital. I got admitted, and they said I had pneumonia. After that, they tested my sputum, and I was told I had TB.”(3^rd^ female FGD).

Even when patients sought health care at the Sub-County or the County hospitals this again did not guarantee a timely diagnosis. Some participants who sought treatment from the hospitals reported having been tested for TB, tests coming back as negative, receiving treatment for other conditions and only later being diagnosed as having TB. This was illustrated by the following participants:

“I was tested for TB twice, but it was negative the 3rd time I was confirmed TB positive.” (IDI Female 30 years).

## Discussion

The study found that patients’ health-seeking pathways were diverse. There was no clear pathway to care for TB patients, the participants adopted different pathways and kept changing from one provider to the other. Health-seeking trajectories showed that patients started with services closer to them such as self-medication, herbal remedies, traditional healers, and primary health care facilities and went to the hospital only after the lower level pathways had failed to provide relief from the symptoms. Although TB symptoms were experienced, patients did not connect them to TB, and this resulted in self-medication by purchasing medicine from drug shops. Patients only went to a health facility after experiencing severe symptoms. This is in agreement with other studies that have shown the importance of drug shops and chemists as the first contact of healthcare [13, 23, 35]. Globally, studies have shown that pharmacy and drug shops owners seldom refer patients for specialised medical treatment [36, 37]. The study also revealed that TB patients delayed seeking health care from the health facilities while receiving care from the informal health care providers. Similar findings were documented in Ethiopia [38], Malawi [39], Tanzania [40], Nepal [41] and Ghana [42]. These providers are crucial in the referral system necessary for timely TB diagnosis and should be educated on the importance of referring patients with a prolonged cough for a TB test even as they continue offering their therapy. However, it is noted that even those who sought treatment from the formal health care experienced delays in TB diagnosis.

Most participants commenced care at the primary health care facilities and the private clinics where there were no TB diagnostic services. The findings are in agreement with recent patient-pathway analyses in five countries that showed that more than 40% of TB patients initiated care in low-level facilities that did not have TB diagnostic capacity [43]. In Kenya, most dispensaries are managed by community health nurses and do not offer laboratory services, and therefore a TB diagnosis is not possible. Similarly, private practitioners particularly those in rural areas are inadequately trained and have a limited diagnostic capacity to manage a TB patient, but due to a motivation to generate revenue, they treat and retain the patient repeatedly without referring them to the appropriate facility.

The different treatment pathways resulted in substantial delay due to different factors illustrated by the conceptual framework of factors leading to delay in TB diagnosis. The factors were categorized into three sub-categories namely individual, social-cultural and structural factors.

The myths on cultural explanations of the cause of TB led to alternative approaches such as traditional healers and herbalists as opposed to seeking modern medicine. This resonates with the findings of Nyasulu et al. (2016) who documented that cultural and traditional practices, as well as beliefs in witchcraft as a cause of illness, are major causes of delay in TB diagnosis. Similarly, a study done in rural South Africa found that community members associated breaking cultural norms to be the cause of TB whose remedy was traditional medicine [44]. Such myths and beliefs should be targeted through awareness creation since they often lead to delayed treatment, increased transmission of TB in the community as well as the risk of development of multidrug-resistant strains.

Social-cultural factors such as gender roles were also key in influencing the time taken from the development of symptoms to the time the patient sought health care. Gender refers to the behaviour, expectations and roles within a social, economic and cultural context ascribed to being male or female. Evidence has shown that social construction of gender greatly influences the health-seeking behaviour [45]. In the present study, masculinity was a major source of delay. Both genders confirmed that male patients compared to female patients, do not seek treatment from a health facility. The findings of longer patient delay among males are consistent with those of other studies done in Uganda [46] and South Africa [47], Malawi [48]. This may be a contributing factor to the higher burden of TB among males compared to females. A recent TB survey done in Kenya showed that the prevalence of TB was higher in males at 809 per 100,000 population compared to females at 359 per 100,000[49]. Elsewhere empirical evidence has shown that there is a higher burden of TB among males which may be as a result of the interplay between a true higher incidence of the disease among men and their poor interaction with primary health care facilities as well as long delays in seeking treatment for various illnesses including TB [50]. In the Pokot community, men viewed themselves as strong and seeking treatment promptly was viewed as an act of weakness. This form of masculinity where men are viewed as without emotions and lacking vulnerability is culturally embedded. It leads to delay in men seeking health care and as a result being underserved by the health care system [48, 51, 52]. Evidence has shown that men are more likely to have a delayed TB and HIV diagnosis and to die during treatment [53–56]. Economic factors in nomadic pastoralist communities are also likely to disadvantage men accessing to the healthcare and thus cause longer delays in TB diagnosis amongst men. Chikovore et al. (2015) rightfully argue that, unless healthcare system adopts a more male inclusive approach, they will continue to serve as a reservoir for TB transmission in the community.

Finally, structural factors such as inadequate diagnostic capacity, distant facilities and lack of finances resulted in a delayed diagnosis. Poor diagnostic capacity led to misdiagnosis which was a hindrance to timely diagnosis. This could be because the standard diagnostic techniques for TB in Kenya is by sputum examination and cultures which are not sensitive enough to capture disease in the early stages. Since the discovery of TB in 1882 sputum microscopy has remained the cornerstone for TB diagnosis in low and middle-income countries [57]. Although sputum microscopy is economic and convenient, it has low sensitivity and only detects cases with high bacterial load and advanced cases [57–60]. Chest X-ray offers a fast and sensitive test for diagnoses of TB. However, it requires specialized manpower and therefore not available in the primary health care facilities. More sensitive tests such as radiography, a molecular diagnosis that can capture TB cases early enough are expensive and inadequate.

In many studies factors affecting delay in TB diagnosis have been dichotomized as patient factors or health system factors [2, 3, 61] with factors such as distant facilities and lack of finances being categorized as patients’ factors [62, 63]. However, in the current study distant facilities and lack of finances were conceptualized as structural factors where the health care system should be the point of intervention. Currently in Kenya, TB treatment is offered for free but patients encounter a lot of hidden cost through transport and diagnostic tests.

The strength of this study is in the use of two methods of data collection. While IDIs allowed for exploration of the detailed experience of each patient health-seeking pathway, the FGDs were helpful in gathering rich discussion on the cultural beliefs surrounding TB diagnosis and treatment. FGDs allowed for a wider scope of understanding as the participants facilitated each other in the discussions [64]. One limitation of the study is the use of IDIs to study patients’ lived experiences of seeking TB diagnosis and treatment. The method was not sufficient to yield a detailed account of the patients’ experiences. A method such as ethnography which does not focus on reports about activities but is driven by the interest of being there and observing events as they occur [65] would have been more appropriate. Secondly, the study findings are based on a sample of patients who were eventually able to access treatment. This excludes the views of TB patients who may have experienced delays and were unable to access TB treatment.

## Conclusion

This study showed that pathway to TB diagnosis and treatment was long and frustrating and caused patients’ suffering. Approaches geared towards timely diagnosis are recommended to avoid the consequences of the pathway of delay in TB diagnosis which includes increased disease transmission. There is a need to improve timely diagnosis through scaling up diagnostic capacity. Similarly, the health system should decentralise TB services as much as possible to increase accessibility and offer free diagnostic services. The TB program should also scale up health education to improve TB awareness in the community. Innovative approaches such as mobile clinics as well as intensive case finding should be incorporated in the TB program in the pastoralist communities.

## Supporting information

S1 Text In-depth interview and focus group discussion guides. (DOCX)
