# Supplementary material for: Health-seeking pathway and factors leading to delays in tuberculosis diagnosis in West Pokot County, Kenya: A grounded theory study
Source: PLoS One. 2018 Nov 28;13(11):e0207995. doi: 10.1371/journal.pone.0207995 (PMC6261612; doi:10.1371/journal.pone.0207995)
Supplement: S1 Text — (DOCX) [file pone.0207995.s001.docx]

**S1 Text. In-depth interview and focus group discussion guides**

**In-depth interview guide in Kiswahili language**

1. Nieleze kwa kina uliyoyapitia baada ya kupata ugonjwa wa kifua kikuu. ( Jinsi ulivyo kuja kushuku ulikuwa na ugonjwa wa kifua kikuu, hatua ulizozichukua kutafuta matibabu, uliyoyapitia kabla ya utambuzi wa ugonjwa, Ilikuchukua muda gani kujua ulikuwa unaungua kifua kikuu,
2. Kwa wa nini watu huchukua muda kabla ya kwenda hospitalini wanapo pata dalili za kifua kikuu? (Kwa upande wako nini kilikuzuia kupata matibabu ya kifua kikuu kwa haraka?
3. Katita kijiji hiki kuna matibabu ya kiasiri ya kutibu kifua kikuu?
4. Ulihisi namna gani ulipogundua ulikua na ugonjwa wa kifua kikuu?
5. Msaada gani ulipata kutoka kwa familia, marafiki, jamii, majirani au wafanyakazi wa afya?
6. Ulikubana na unyanyapaa kabla, wakati wa, na baada ya matibabu?
7. Kuna taarifa yoyote ingine kuhusu kuishi na ugonjwa wa kifua kikuu ungependa kuniambia?

**In-depth interview guide (English translation).**

1. Tell me about the experience you have gone through since you started ailing from Tuberculosis (TB)? Probes: When did you come to suspect that you had TB? What actions did you take when you started experiencing TB symptoms (the health seeking practices in chronological order)?” How long did you take from symptom onset to diagnosis?
2. Why do people delay before seeking TB treatment from the health facility? Probe: What hindered you from accessing TB treatment promptly?
3. What traditional forms of treatment for TB are available in this community?
4. How did you feel to realise you had TB?
5. How did those around you treat you when they knew you had TB? Probe on social support system from family, friends, the community, the neighbours or health workers/facilities.
6. Tell me about any form of discrimination/stigma you may have experienced before, during and after treatment? Probes: Why do you think people discriminate against TB patients?
7. Is there any other information regarding your experience with TB you would want to tell me?

**Focus group discussion guide in Kiswahili language**

1. Mtu anapoungua huu ugonjwa wa kifua kikuu, anapitia yapi katika hii jamii?
2. Je, wagonjwa wa kifua kikuu hutafuta matibabu mara moja kutoka kituo cha afya wanaposhuku wako na ugonjwa wa kifua kikuu?
3. Aina gani nyingine ya huduma za afya ambazo mgonjwa wa kifua kikuu hutafuta? (dawa za jadi / dawa za kienyeji au tiba ya ugojwa wa kifua kikuu)
4. Je, kuna maelezo gani ya kiasiri kuhusu kiini cha kifua kikuu? (tamaduni kuhusu kifua kikuu katika jamii)
5. Je, wagonjwa wa kifua kikuu hutegwa au hujitenga wenyewe?
6. Msaada gani wa kijamii unaotolewa kwa wagonjwa wa kifua kikuu katika familia/jamii yako?
7. Je, kuna mengine kuhusu ugonjwa wa kifua kikuu katika jamii mngependa kueleza?

**Focus group discussion guide (English translation)**

1. Tell me about the experience TB patients go through to get TB diagnosis and treatment
2. Do TB patients seek treatment promptly from the health facility immediately they suspects they have TB? Why?
3. What other forms of health care for TB patients is available in this community? Probes: Traditional medicine/herbs or treatment for TB available in the community
4. What are some of the traditional explanations to the causes of TB in this community? (Any cultural beliefs related to TB?)
5. Do TB patients face any form of discrimination (do they isolate themselves or are they isolated by others? Why?
6. What kind of social support do TB patients experience in this community?
7. Is there any other issue regarding TB in the community that you would wish to share with us?
